# ABC Efflux Transporters and the Circuitry of miRNAs: Kinetics of Expression in Cancer Drug Resistance

**DOI:** 10.3390/ijms21082985

**Published:** 2020-04-23

**Authors:** Bruno C. Gomes, Mónica Honrado, Ana Armada, Miguel Viveiros, José Rueff, António S. Rodrigues

**Affiliations:** 1 Centre for Toxicogenomics and Human Health; Genetics, Oncology and Human Toxicology, NOVA Medical School, Universidade NOVA de Lisboa, Rua Câmara Pestana 6, 1150-008 Lisbon, Portugal; bruno.gomes@nms.unl.pt (B.C.G.); monica.honrado@nms.unl.pt (M.H.); jose.rueff@nms.unl.pt (J.R.); 2 Global Health and Tropical Medicine, Instituto de Higiene e Medicina Tropical, Universidade NOVA de Lisboa, Rua da Junqueira 100, 1349-008 Lisbon, Portugal; aarmada@ihmt.unl.pt (A.A.); MViveiros@ihmt.unl.pt (M.V.)

**Keywords:** cancer drug resistance, microRNAs, gene regulation, ABC drug transporters, ABCB1, MDR1, doxorubicin

## Abstract

Cancer drug resistance (CDR) is a major problem in therapeutic failure. Over 90% of patients with metastatic cancer present CDR. Several mechanisms underlie CDR, including the increased expression of efflux ABC transporters and epigenetic phenomena. Nevertheless, a topic that is not usually addressed is the mechanism underlying the loss of CDR once the challenge to these cells is withdrawn. A KCR cell line (doxorubicin-resistant, expressing ABCB1) was used to induce loss of resistance by withdrawing doxorubicin in culture medium. ABCB1 activity was analysed by fluorescence microscopy and flow cytometry through substrate (DiOC2) retention assays. The expression of 1008 microRNAs was assessed before and after doxorubicin withdrawal. After 16 weeks of doxorubicin withdrawal, a decrease of ABCB1 activity and expression occurred. Moreover, we determined a signature of 23 microRNAs, 13 underexpressed and 10 overexpressed, as a tool to assess loss of resistance. Through pathway enrichment analysis, “Pathways in cancer”, “Proteoglycans in cancer” and “ECM-receptor interaction” were identified as relevant in the loss of CDR. Taken together, the data reinforce the assumption that ABCB1 plays a major role in the kinetics of CDR, and their levels of expression are in the dependence of the circuitry of cell miRNAs.

## 1. Introduction

Cancer drug resistance (CDR) is a main challenging issue in advanced and metastatic tumors. Even in targeted therapy, resistance mechanisms are frequently triggered to circumvent therapy involving fluctuations in the activation of signaling pathways downstream to the receptors to targeted therapies called compensatory adaptation or oncogenic bypass.

Cancer drug resistance encompasses endogenous and acquired drug resistance, and the underlying mechanisms are yet to be fully elucidated in all their features and mechanisms [1]. Increased drug efflux, target mutations, alterations in cell cycle checkpoints, the inhibition of apoptosis, and increased DNA damage repair have been identified in drug resistance [2,3,4,5]. Among the mechanisms that mediate drug resistance, the most widely studied mechanism is drug efflux through the membrane, given that tumor cells overexpress drug transport proteins located in the cytoplasmic membrane, including the ATP-binding cassette (ABC) transporters P-gp (P-glycoprotein, ABCB1), multidrug resistance-associated protein 1, ABCC1, and breast cancer resistance protein, ABCG2 (BCRP) [6,7].

Several attempts to develop inhibitors of ABC transporters have been pursued over the years. Three generations of ABCB1 inhibitors have been examined in preclinical and clinical studies. However, those trials have largely failed to demonstrate an improvement in the therapeutic efficacy of antitumor agents, essentially due to systemic toxicity attributed to the inhibition of ABC transporters [8]. Therefore, new and innovative strategies need to be developed, to identify and overcome CDR mechanisms.

Acquired CDR is a time-dependent process. Therefore, one means of identifying the onset of CDR in patients is the sequential sampling of tumor tissue, although this is only feasible in liquid cancers (blood cancers, leukemia) where blood samples can be harvested to monitor resistance to therapy [9]. In solid tumors, this approach is hardly attainable, thus, other strategies are required to identify a signature of CDR.

In recent years, microRNAs (miRNAs) have been proposed as biomarkers of CDR [10,11,12,13]. These are small noncoding RNAs that regulate gene expression by post-transcriptionally targeting the 3′-UTR region of mRNA. They act as master regulators of protein expression by blocking translation and forming a circuitry of epigenomic gene expression [14]. Due to their small size (22–25 nucleotides), miRNAs can bind to several different mRNAs, while the same mRNA can be targeted by several miRNAs [15].

More than two thousand human miRNAs have been identified thus far [16]. To date, several studies have shown that drug resistance is influenced by miRNAs [17,18,19,20,21]. Various miRNAs have been reported to have a direct or indirect role in the regulation of the expression [17,22,23] or activity [24] of ABCB1 in CDR. Several groups have developed cell lines resistant to specific cancer drugs, such as doxorubicin, or paclitaxel, to study biomarkers of CDR [25,26]. However, the time course of occurrence of resistance mechanisms has not always been possible to assess and is not yet generally known and understood [9,27,28]. Furthermore, another issue that has not been addressed is the mechanism underlying the loss of resistance to therapy once the challenge to these cells is withdrawn. Therefore, we have used the KCR cell line, a cell line with acquired resistance to doxorubicin due to the overexpression of ABCB1 [26], to assess the loss of CDR. These cells are cultured with doxorubicin to maintain the expression of ABCB1 and resistance. Thus, in order to assess the loss of resistance, cells were cultured without DOX for 16 weeks. Cell viability was monitored through colorimetric assays, ABCB1 expression through real-time PCR and western blot, and finally, functional accumulation assays were performed by flow cytometry and fluorescence microscopy to verify ABCB1 activity. Then, we profiled 1008 miRNAs through real-time PCR, comparing the KCR cell line at different time points through loss of DOX resistance. The efflux activity of ABCB1 in KCR cells was significantly reduced with loss of DOX resistance and we identified a signature of microRNAs expression that might be a good tool to assess loss of drug resistance.

## 2. Results

### 2.1. Loss of Doxorubicin Resistance Induces a Lower Cell Viability

**Figure 1:**
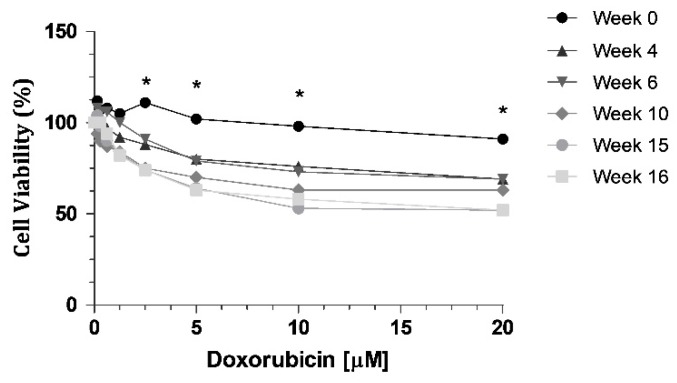
MTT assay showing a decrease in cell viability when KCR cells were treated with DOX at different time points for 72 h. Statistical analysis was performed based on 2-way ANOVA with Bonferroni’s post-test. *: Statistical significance was considered when *p* < 0.05.

Cell resistance to DOX was evaluated at different time intervals (week 0, 4, 6, 10, 15 and 16) after DOX withdrawal, using the MTT assay, with eight different concentrations (0.15; 0.3; 0.6; 1.25; 2.5; 5; 10 and 20 μM). We can observe a decrease of cell viability through time (Figure 1), indicating a loss of resistance to DOX. When we compared week 0 with all the other weeks tested, there were statistically significant decreases at week 10, week 15 and week 16 (Figure 1). The highest concentration of DOX, 20 µM, led to a 39% decrease in cell viability of KCR cells without DOX at week 16, compared to parental KCR cells (week 0).

### 2.2. Loss of Doxorubicin Resistance is Accompanied by a Decrease in ABCB1 Activity

The increase in ABCB1 expression has long been known to correlate with drug resistance in cancer. The KCR cell line at time zero (week 0), overexpresses ABCB1 efflux pumps which are responsible for the drug resistance observed in this cell line. To evaluate the efflux activity of ABCB1, the DiOC_2_ efflux assay was performed as previously described [20,29] and VP, a synthetic known inhibitor of ABCB1, was used to block efflux.

**Figure 2:**
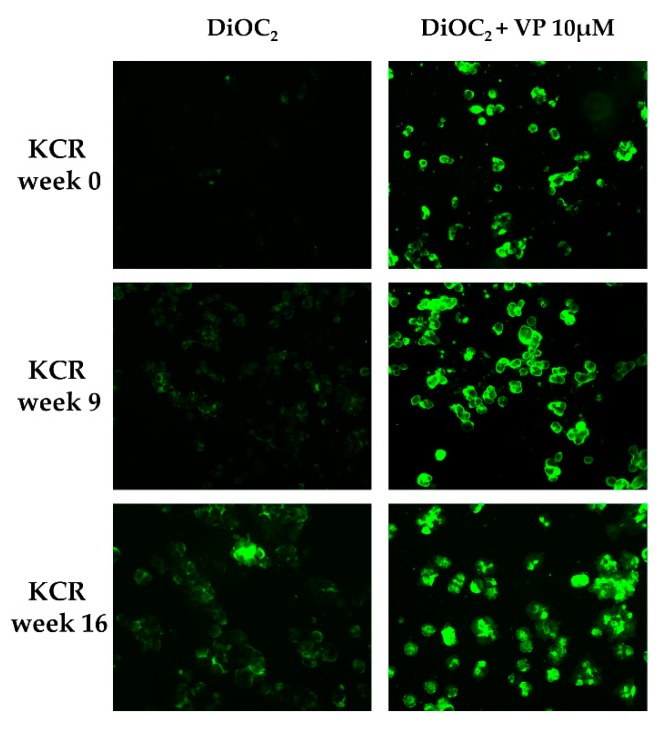
ABCB1 efflux activity verified by fluorescent microscopy (200× magnification) after treatment with DiOC_2_ and verapamil (VP) as a positive control. In the figure, we can observe an accumulation of DiOC_2_ inside the cells over time, indicating that cells decrease ABCB1 efflux activity. Green color reflects the accumulation of DiOC_2_ inside the cell.

**Figure 3:**
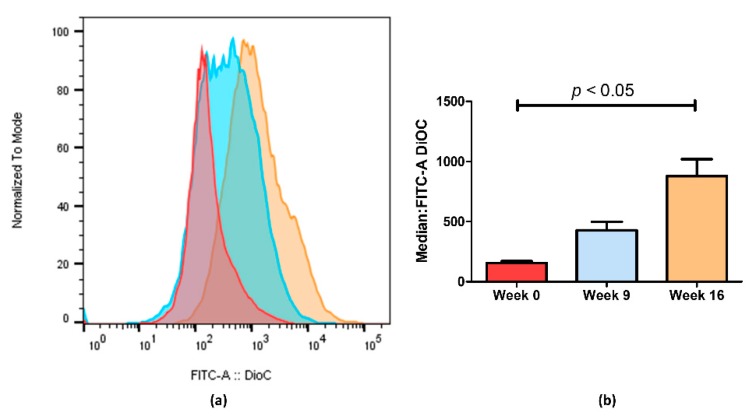
ABCB1 efflux activity measured by flow cytometry after treatment with DiOC_2_. (**a**) Representative fluorescent intensity histogram in week 0 (red), week 9 (blue) and week 16 (brown). We can see an increase in fluorescent intensity as time passes, indicating that less ABCB1 membrane transporters are active in week 16. In (**b**), we show the mean results of two independent assays. We can observe a 5.6-fold decrease between week 0 and week 16. Data are expressed as the median ± SEM. Statistical analysis was done by using one-way analysis of variance and Bonferroni’s multiple comparison test. *p*-value <0.05 was considered statistically significant.

DiOC_2_ efflux was assessed through fluorescence microscopy and flow cytometry in KCR cells on week 0, 9 and 16, by exposing them to DiOC_2_ with or without inhibitor (Figure 2 and Figure 3). KCR cells without VP (Figure 2 (left)) displayed an increase of fluorescence compared to week 9 and a more significant increase on week 16 compared to parental KCR cells (week 0), indicating a higher efflux of DiOC_2_. Therefore, KCR cells lose the activity of ABCB1 efflux pumps as time passes. No difference was observed in the presence of VP (Figure 2 (right)), indicating an inhibition of ABCB1 membrane transporters.

Through flow cytometry, we could quantify the difference observed by fluorescence microscopy imaging (Figure 3). Figure 3a shows an increase in fluorescence on KCR week 9 and a significantly higher increase on week 16 compared with the parental KCR cells (week 0). Since more accumulation indicates less efflux of ABCB1 efflux transporter, these results further indicate that the efflux activity of ABCB1 in KCR cells was significantly reduced on week 16 compared with KCR week 0. Figure 3b shows the results from Figure 3a expressed as the median ± SEM and showing the statistical difference between KCR week 0 and KCR week 16.

### 2.3. ABCB1 is Downregulated in KCR Cells without Doxorubicin for 16 Weeks

The expression of ABCB1 was evaluated in cell lysates of KCR weeks 0, 10 and 15 through Western blot, and in KCR weeks 0 and 16 through real-time qPCR.

**Figure 4:**
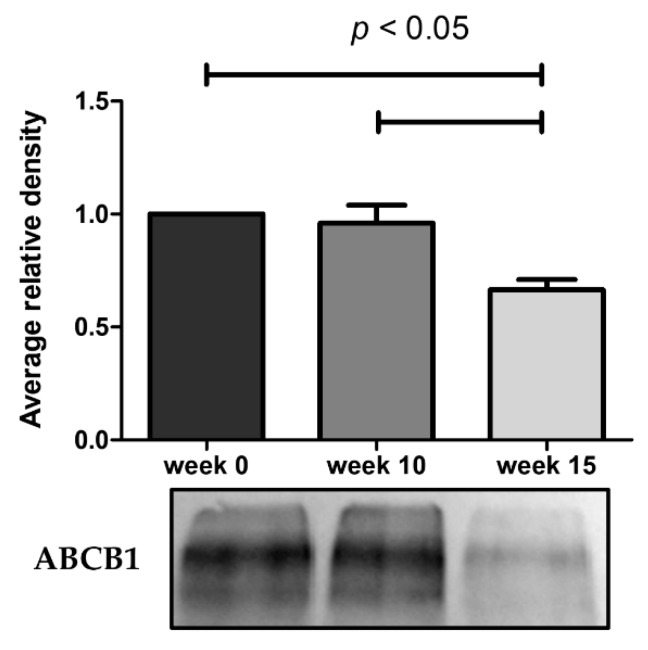
Western blot analysis for protein expression of ABCB1 in KCR cells in week 0, 10 and 15. Values are means of two independent experiments expressed as the mean ± SEM. Data was normalized against total protein determined by densitometric analysis using Image J. We can observe a decrease of ABCB1 protein expression with time. Statistical analysis was done by using one-way analysis of variance and Bonferroni’s multiple comparison test. *p*-value < 0.05 was considered statistically significant.

**Figure 5:**
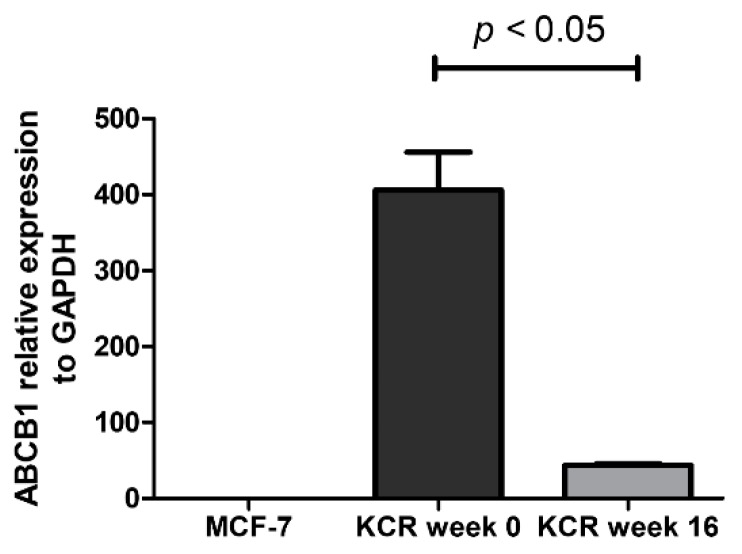
Relative expression of ABCB1 gene assessed by real-time qPCR. The values represent three independent experiments using the 2^−(ΔCt)^ method. ABCB1 expression was normalized to the housekeeping gene GAPDH. We can observe a 9.3-fold decrease of ABCB1 mRNA expression between KCR weeks 0 and 16. ABCB1 expression in MCF-7 cells is negligible. Statistical analysis was performed using unpaired T-test. *p* < 0.05 was considered statistically significant.

As illustrated in Figure 4, the ABCB1 protein expression undergoes a marked decrease at week 15. The results obtained from these assays demonstrate that the reduced efflux activity of ABCB1 efflux pump observed by flow cytometry and microscopy is due in part to a lower expression of ABCB1 pumps. By RT-qPCR, we also observe a 9.3-fold decrease of ABCB1 mRNA expression in KCR week 16 compared to KCR week zero (Figure 5).

### 2.4. miRNAs are Differently Expressed in KCR Cell Line with Time

In order to identify differentially expressed miRNAs after 16 weeks without DOX, we quantified the relative expression of 1008 miRNAs of parental KCR cells (week 0) and KCR week 16 cells. Table 1 shows the miRNAs with a fold-change greater than 2. Twenty-three miRNAs were differentially expressed in the KCR cells after 16 weeks without DOX. Of these, thirteen miRNAs were underexpressed, while 10 miRNAs were overexpressed.

**Figure 6:**
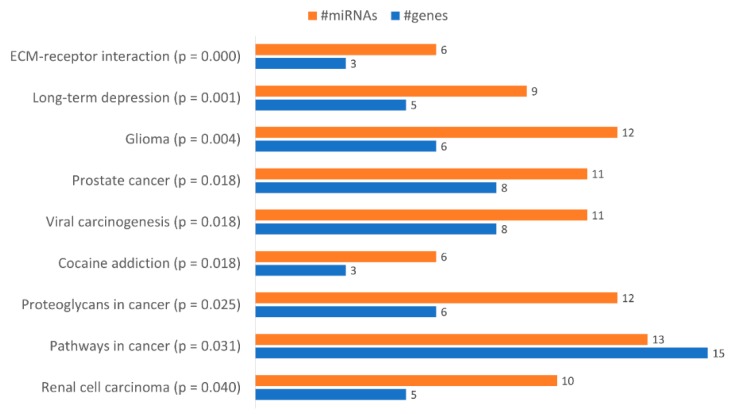
Gene enrichment analysis using KEGG pathways with genes targeted by the differentially expressed miRNAs between KCR week 0 and KCR week 16. Pathways are ordered by increasing *p* value.

With the aim of identifying functional pathways associated with the miRNAs in Table 1, we used DIANA-mirPath, a miRNA pathway analysis webserver that allows an enriched analysis of KEGG database of molecular functions’ categories and GO terms. Thus, the KEGG categories identified using all miRNAs differently expressed were “Renal cell carcinoma”, “Pathways in cancer”, “Proteoglycans in cancer”, “Prostate cancer”, “Viral carcinogenesis”, “Cocaine addiction”, “Glioma”, “Long-term depression” and “ECM-receptor interaction” (Figure 6). Only the categories “Pathways in cancer”, “Proteoglycans in cancer” and “ECM-receptor interaction” were considered relevant to drug resistance. The category “Pathways in Cancer” has the highest number of genes identified (15), being regulated by 13 miRNAs out of 23 (Table 2). In “Proteoglycans in cancer”, 6 target genes were identified, being regulated by 12 miRNAs (Table 3). In “ECM-receptor interaction”, 3 target genes were identified, being regulated by 6 out of 23 miRNAs (Table 4).

**Figure 7:**
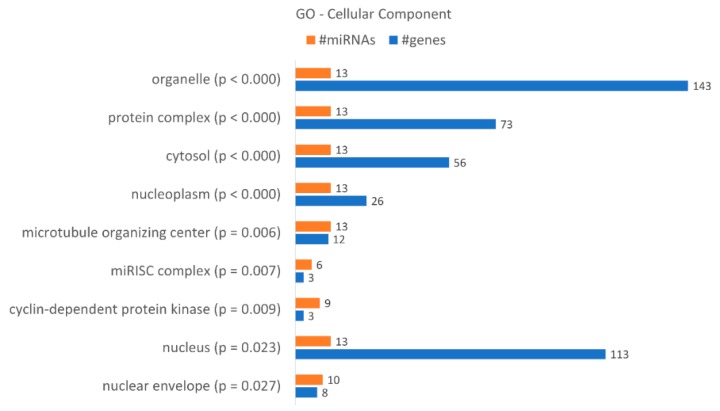
Gene enrichment analysis using GO “Cellular Component” terms, with genes targeted by the differentially expressed miRNAs between KCR week 0 and KCR week 16. Cellular component terms are ordered by increasing *p* value.

A gene ontology enrichment analysis showed that KCR week 16 has 9 “cellular components” terms associated with the dysregulated miRNAs and loss of resistance (Figure 7), with a higher number of target genes identified in the “organelle”, “nucleus”, “protein complex” and “cytosol”. Although they only have three target genes, we would like to highlight the “microRNA-RISC complex”, a known complex involved in miRNA dependent gene regulation. The putative targets are DICER1, AGO3 and AGO1, central to the action of miRNAs.

**Figure 8:**
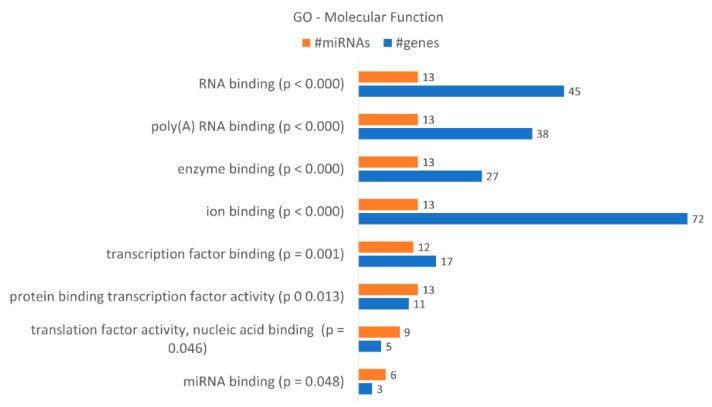
Gene enrichment analysis using GO “Molecular Function” terms, with genes targeted by the differentially expressed miRNAs between KCR week 0 and KCR week 16. Molecular function terms are ordered by increasing *p* value.

These data correlate with the number of differentially expressed miRNAs. DICER1 is putatively regulated by hsa-miR-34a-5p, hsa-miR-877-5p, hsa-miR-342-3p, hsa-miR-1207-5p, hsa-miR-183-3p and hsa-miR-502-5p, as indicated by bioinformatics analysis, and AGO3 by hsa-miR-34a-5p, hsa-miR-183-3p and hsa-miR-502-5p. AGO1 is putatively regulated by hsa-miR-34a-5p, hsa-miR-877-5p, hsa-miR-183-3p and hsa-miR-502-5p. A gene enrichment analysis performed according to “molecular function” (Figure 8) revealed that the dysregulated miRNAs found in KCR week 16 regulate 8 “molecular functions” terms, in particular “ion binding” with 72 putative targets, “RNA binding” with 45, “poly(A) RNA binding with 38 and “enzyme binding” with 27 targets. Although less genes were detected, we would like to highlight the molecular function “miRNA binding” with three putative targets, the same as detected in “microRNA-RISC complex”. We also enriched our data according to “biological process” terms, revealing 57 different terms (Table 5). The highest number of genes were detected in “cellular nitrogen compound metabolic process”, with 79 genes, “biosynthetic process”, with 60 genes, and “response to stress”, with 46 genes.

## 3. Discussion

Cancer drug resistance affects millions of cancer patients worldwide and is responsible for treatment failure in over 90% of patients with metastatic cancer. Understanding the molecular mechanisms underlying drug resistance may aid the design of novel strategies to overcome CDR. Several molecular pathways are directly involved in acquired drug resistance, including increased expression and activity of efflux ABC transporters and epigenetic phenomena underlying the levels of ABC proteins [30]. In vitro cell culture models have identified a number of mechanisms of CDR, of which many, if not all, may contribute to cancer resistance in the clinical setting [9,20,31,32].

In order to overcome CDR, some therapeutic strategies suggest the intermittent management of cancer drugs, whereupon drugs are applied in time cycles to prevent acquired resistance [33,34]. However, as acquired CDR is a time-dependent process, the assessment of the mechanisms that are activated as cells to develop resistance is critical. One interesting phenomenon that we were able to identify in a model of chronic myeloid leukemia was the sequential activation of different ABC transporter expressions (ABCB1, ABCG2, ABCC1), as cells gained resistance to the tyrosine kinase inhibitor Imatinib [9], and the relatively unstable pattern of expression of these transporters in human samples [28]. These observations indicated that the time-dependent analysis of CDR is critical to a better understanding of the mechanisms involved. The large majority of studies address gain of drug resistance in order to develop drugs that could overcome resistance. In contrast, a topic that is not commonly addressed is the mechanism underlying the loss of resistance to therapy once the challenge to these cells is withdrawn. A loss of resistance may involve the inactivation of the same mechanisms activated in gain of drug resistance. Thus, our aim was to understand if gain of drug resistance is a mechanistic mirror image of loss of drug resistance.

For this purpose, we used a DOX-resistant subline of the human breast adenocarcinoma cell line MCF-7, KCR, which is characterized by overexpressing ABCB1. These cells are cultured with doxorubicin to maintain the expression of ABCB1 and some resistance. The loss of DOX resistance was induced by withdrawing DOX. Our findings revealed that after a withdrawal of DOX for 16 weeks, there was a 9.3-fold decrease in ABCB1 expression, and a 40% decrease in protein expression. This decrease in ABCB1 expression was also validated by a decrease in ABCB1 efflux activity of 5.6-fold, when comparing week 0 and week 16. The sensitivity to DOX at week 10 is increased compared to week 0 in the MTT viability assay, although the levels of ABCB1 do not seem to drop significantly. Nevertheless, the cytometry data indicate a reduction in efflux at week 9, although with a broader peak, in contrast to week 16, where the cytometry peak is narrower. This indicates that we have a more heterogeneous population of cells at week 9 than week 16. The cell viability results are more concordant with the cytometry data (which indicate global efflux activity), than with the loss of ABCB1 protein alone. This also suggests that other proteins, e.g., ABCG2, ABCC1, could be involved in the efflux of DOX. It is possible that these efflux proteins drop more rapidly in the first weeks compared to ABCB1. Nevertheless, in another study, the KCR cell line was found to express 72 000-fold more ABCB1 mRNA levels compared to the parental cell line, whereas that of MRP1/ABCC1 was only elevated sevenfold [35]. This suggests that ABCB1 is the major efflux protein in this cell line. ABCG2 and other efflux proteins were not assessed in this work.

Several biomarkers have been associated with CDR, namely miRNAs [14,30]. Biomarkers are urgently needed to identify upfront patients who are developing CDR. In this study, we found 23 miRNAs that were differentially expressed in the KCR cells after losing drug resistance, 13 of them underexpressed, and 10 miRNAs overexpressed. Some of these miRNAs have been reported as regulators of cellular pathways associated with control of cell growth, proliferation, differentiation and survival. After enrichment analysis using KEGG pathways, the pathways “Pathways in cancer”, “Proteoglycans in cancer” and “ECM-receptor interaction” were identified as having a bigger impact in drug resistance. The 23 miRNAs differently expressed target 15, 6 and 3 genes in these pathways, respectively. All of these miRNA:gene interactions were experimentally validated, as we used TarBase for the enrichment analysis [36].

Of the miRNAs identified in “Pathways in cancer”, 13 were predicted to target 15 genes involved in survival and apoptotic pathways. In total, 9 miRNAs of the 13 are overexpressed in KCR cells sensitive to DOX. Thus, proliferative and anti-apoptotic genes (BCL-2, E2F3, GNAS, CTNNB1, etc.) are potential targets of these miRNAs. In agreement, we observed that KCR week 16 cells are less viable after treatment with DOX, as observed in the MTT assay. These results are expected, as proliferation and survival are drug resistance phenotypes, and KCR cells lost drug resistance after 16 weeks. We did not assess apoptosis nor cell cycle arrest, but these assays are programmed in future studies.

Previous studies have already reported an interaction between these miRNAs and these genes. For instance, Tang et al. showed that miR-1307-3p acts as a tumour suppressor inhibiting the expression of the BCL2 protein in colorectal carcinogenesis cell lines [37]. However, Han et al. show that miR-1307-3p in MCF10A stimulated cell proliferation [38].

Other miRNAs from this pathway that are of interest are the miRNAs that are underexpressed (hsa-miR-34a-5p, hsa-miR-155-3p, hsa-miR-877-5p, hsa-miR-3691-5p). The last two have never been reported in drug resistance and the first two have been reported as tumor suppressors and proven to reverse drug resistance [39,40,41,42]. However, Pu et al. showed that miR-34a-5p is up-regulated in multi-chemoresistant cell lines compared to the multi-chemosensitive osteosarcoma cell lines [43], as we observe in our cells. A possible explanation for the discrepancies is that these miRNAs might play multi-functional roles by targeting different genes and might also play distinct roles in different types of cells. More studies are needed to elucidate the regulation mechanism of these miRNAs in drug resistance.

Future work is planned to ascertain the role of the individual miRNAs identified CDR. As a whole, this work tends to suggest that ABC transporters’ family are subject to microRNA- mediated gene regulation and the data here described is planned to assess if the miRNA signature found is representative of drug resistance in general.

## 4. Materials and Methods

### 4.1. Cell Culture

The KCR cell line was kindly provided by Professor Joseph Molnar, Szeged Foundation for Cancer Research, Hungary (http://www.szegedirakkutatasert.hu/collaboration.html). The KCR cell line is a DOX-resistant subline of human breast adenocarcinoma cell line MCF-7 and resistance is maintained by culturing the cells in the presence of 1 µM DOX [26]. KCR cells were cultured in RPMI 1640 medium from Gibco (USA), with 10% foetal bovine serum (FBS) and 1% penicillin-streptomycin (with 10,000 units penicillin and 10 mg streptomycin per mL), both from Sigma-Aldrich(Darmstadt, Germany). Culture medium was replaced three times a week and sub-cultured by trypsinization (10% trypsin in EDTA solution; Sigma-Aldrich) when confluence reaches approximately 80%. The KCR cell line was incubated at 37 °C in a humidified 5% CO_2_ chamber. In order to lose DOX resistance, KCR cell line was cultured without DOX for 16 weeks.

### 4.2. Cell Viability (MTT Assay)

Cell viability was assessed using the methylthiazolyldiphenyl-tetrazolium bromide (MTT) assay (Sigma-Aldrich, Darmstadt, Germany). Approximately 10,000 cells/well were cultured in complete medium in 96-well plates. The cells were allowed to grow for 24 h. Next, different concentrations of DOX (MEDAC, Hamburg, Germany) were added to the cells (0; 0.15; 0.3; 0.6; 1.25; 2.5; 5; 10 and 20 μM), and allowed to grow for 72 h. Then, MTT was added to each well at a final concentration of 0.5 mg/mL. Cells were then incubated for 4h. Next, a solution of sodium dodecyl sulphate (10% SDS in 0.01 M HCl, (Sigma-Aldrich, Darmstadt, Germany) was added to dissolve formazan crystals and incubated overnight. Absorbance was read at 570 nm in a microplate reader SpectraMax i3x. This assay was performed for KCR cells over time, as the selective pressure to maintain drug resistance was withdrawn, specifically at 0, 4, 6, 10, 15 and 16 weeks.

### 4.3. Evaluation of ABCB1 Activity by Fluorescence Microscopy

ABCB1 activity was measured by fluorescence microscopy as previously described, with minor modifications [20]. Briefly, cells were seeded on sterilized glass coverslips in 24-well culture plates at a density of 2 × 10^5^ cells/mL and allowed to grow overnight. The following day, medium was removed from wells, and fresh medium with and without 10 µM verapamil (VP; Sigma-Aldrich, Darmstadt, Germany) was added to each duplicated experiment well and incubated for 30 min before addition of 1 µg/mL of DiOC_2_ (Molecular Probes^TM^, Eugene, OR, USA) for 1 h at 37 °C. Subsequently, the medium was removed, and dye free medium was added with the presence or absence of VP for another 1 h. Slides were rinsed twice in cold PBS and then cells were examined using a fluorescence microscopy. Cells were examined at 485 nm excitation laser and 530/30 nm emission filter (green fluorescence). This experiment was done using KCR parental cell line (week 0) and KCR with DOX withdrawal for 9 and 16 weeks.

### 4.4. Evaluation of ABCB1 Activity by Flow Cytometry

Flow cytometry was performed according to [20], with minor modifications. Briefly, cells were harvested and placed in 1.5 mL tubes, at a concentration of 2 × 10^6^ cells/mL and pre-incubated in medium (control cells) or treated with 10 µM VP, for 30 min at 37 °C. Then, DiOC_2_ (1 µg/mL) was added and cells were incubated for 1 h at 37 ºC. Later, cells were centrifuged for 10 min at 2000 rpm, and to keep efflux pumps inhibited, cells were then resuspended in 500 µl of fresh medium in the presence or absence (control cells) of VP, and incubated for another hour at 37 °C. Cells were then washed twice with cold PBS, resuspended in 500 µL cold PBS and fluorescence retention was measured by flow cytometry, using FACS CANTO II^TM^ (BD Biosciences, Franklin Lakes, NJ, USA), at an excitation wavelength of 488 nm, and the emission was recorded using 525/40 BP filters (FITC-A channel). Data were collected for at least 10,000 events per sample and analyzed using FlowJoTM10 software (FlowJo™ Software for Windows Version 10. Ashland, OR, USA). This experiment was done using KCR parental cells (week 0) and KCR with DOX withdrawal after 9 and 16 weeks.

### 4.5. ABCB1 Protein Expression by Western BLOT

#### 4.5.1. Purification of Membrane Proteins

Cells were harvested by trypsinization, and membrane proteins were isolated by using the Mem-PERM Membrane Protein Extraction Kit according to the manufacturer’s instructions (Thermo Fisher Scientific, Waltham, MA, USA). Briefly, 5 × 10^6^ cells were washed twice with cell wash solution and centrifuged for 5 min at 300× *g*. Then, cells were resuspended in permeabilization buffer and incubated 10 min at 4 °C with constant mixing. The permeabilized cells were centrifuged for 15 min at 16,000× *g*. A solubilization buffer was added to pelleted cells and resuspended by pipetting up and down. Then, cells were incubated at 4 °C for 30 min with constant mixing. Finally, cells were centrifuged at 16,000× *g*, for 15 min at 4 °C, and the supernatant was collected and stored at −80 °C.

#### 4.5.2. Western Blot

Prior to Western blotting, membrane proteins were quantified using the Bradford method (Bio-Rad, Hercules, CA, USA). A bovine serum albumin (BSA) solution of 2 mg/mL (Bio-Rad) was used to prepare a calibration curve, with different dilutions. Our samples (KCR week 0, 10 and 15) were diluted in 1:400 ratio in deionized water. Samples’ absorbance were read at 590 nm (Biotrak II Plate reader, GE Healthcare Life Sciences, Freiburg, Germany).

For the Western blot, protein samples were denatured in Laemmli buffer 2× (4% (*w/v*) SDS 10%; 20% (*v/v*) glycerol 50%; 0.02% (*w/v*) bromophenol blue; 125 mM Tris-HCl pH 6.8; and 10% (*v/v*) 2-Mercaptoethanol), in a proportion of 1:1 and heated at 95 °C for 5 min. Samples were then loaded into a 10% sodium dodecyl sulfate-polyacrylamide gel and subject to electrophoresis at 100 V for 90 min in running buffer 1× (stock solution: 25 mM Trizma-base; 192 mM Glycine; 0.1% (*w/v*) SDS; pH 8.3). The gels were incubated in transfer buffer 1× (stock solution: 25 mM Trizma.base; 192 mM Glycine; 0.1% (*w/v*) SDS; and 10% methanol) for 20 min, as well as the Immun-Blot PVDF membrane (0.2 µm pore size, Bio-Rad, Hercules, CA, USA). Next, proteins were transferred from gel to PVDF membrane in transfer buffer 1× for 60 min at 100 V. Membranes were then blocked using blocking buffer from WesternDot^TM^ 625 Goat Anti-Mouse Western Blot Kit (Invitrogen, Waltham, MA, USA) for 1 h, and then probed overnight at 4 °C with anti-human ABCB1 (dilution 1:1000) (D11: sc-55510, Santa Cruz Biotechnology, Dallas, TX, USA). Then, membranes were washed three times with washing buffer and incubated at room temperature for one hour with anti-mouse secondary antibody (dilution 1:1000) (Invitrogen, Waltham, MA, USA). Finally, the membranes were incubated at room temperature with Qdot^®^ 625 streptavidin for one hour (dilution 1:2000) (Invitrogen, Waltham, MA, USA). The membranes were then visualized under ultra-violet light and captured by ChemiDocTM Touch Imaging System (Bio-Rad, Hercules, California, USA). The protein expression level from Western blot was quantified by densitometry analysis using ImageJ (Rasband, W.S., ImageJ, U.S. National Institutes of Health, Bethesda, MD, USA; imagej.nih.gov). Signal intensities were normalized to total protein by quantifying the intensity of total bands from coomassie blue gel using ImageJ software and background intensity was subtracted.

### 4.6. Total RNA Purification

Total RNA (including miRNAs) from KCR cells was purified with miRNeasy Mini kit (Qiagen, Hilden, Germany), according to the manufacturer’s instructions. Briefly, 6 × 10^6^ cells were harvested and lysed in 700 µL of RLT plus β-mercaptoethanol and conserved at −80 °C until further use. Then, 350 µL of the lysate was diluted in a proportion of 1:2 with Acid Phenol:Chloroform (5:1 Solution pH 4.5; Ambion, Austin, TX, USA), mixed vigorously, and stored for 5 min at room temperature. Next, the lysate was centrifuged for 15 min at ≥12,000× *g* at 4 °C. The aqueous phase was carefully removed and placed in a new tube. After this, 100% ethanol was added in a proportion of 1:1.5 to the aqueous phase and mixed by pipetting. This solution was loaded into an RNeasy Mini column and centrifuged for 15 min at ≥12,000× *g* at room temperature. The flow-through was discarded and the RNeasy Mini column was washed by adding 700 µL of RWT buffer and centrifuged for 15 s at ≥8000× *g*. The flow-through was discarded. A second wash with 500 µL of RPE buffer was done, followed by centrifugation for 15 s at ≥8000× *g*. The flow-through was also discarded. A third wash with 500 µL of RPE buffer was done, followed by a centrifugation for 2 min at ≥8000× *g*. The flow-through was again discarded. Next, a new centrifugation was done for 1 min at ≥8000× *g* at room temperature and 40 µL of nuclease-free water was added directly to the spin column membrane, stored 5 min at room temperature and centrifuged for 1 min at ≥8000× *g* to elute the RNA. This RNA was then stored at −80 °C until further use.

### 4.7. Real-time qPCR Quantification of ABCB1

The relative quantification of ABCB1 mRNA was carried out as published before [20]. Briefly, cDNA was synthesized from 1μg of total RNA using the High Capacity cDNA Reverse Transcription Kits (Applied Biosystems, Foster City, CA, USA) in a final reaction volume of 20 μL, according to the manufacturer’s instructions. For relative quantification, we used a QuantStudio 5 Real-Time PCR System (Applied Biosystems, Foster City, CA, USA) and pre-developed Taqman^®^ primer assays, which were purchased from Applied Biosystems (ABCB1, Hs00184491_m1 and the human GAPDH, 4352934E as the reference gene). All PCR reactions were done in a total volume of 10 μL by using TaqMan^®^ Taqman Universal PCR Master Mix (Applied Biosystems, Foster City, CA, USA). Template controls and reverse transcriptase controls (RT negative) for each cDNA synthesis were included. Thermal cycler conditions were 50 °C for 2 min; 95 °C for 10 min, followed by 40 cycles at 95 °C for 15 s, and at 60 °C for 1 min. The mean values of the triplicate RT-qPCR reactions for each assay were normalized with the expression values for each gene. The relative expression of ABCB1 was performed by the comparative 2^−(ΔΔ*C*t)^ method.

### 4.8. Real-Time qPCR Quantification of miRNAs

The relative expression of 1008 miRNAs in KCR parental cells (week 0) and KCR cells week 16 were quantified by using miScript II RT Kit and Human miRNome miScript^®^ miRNA PCR Array from Qiagen (MIHS-216ZA), in a QuantStudio 5 Real-Time PCR System (Applied Biosystems, Foster City, CA, USA), according to the protocol described by the manufacturer. Briefly, total RNA was converted into cDNA using miScript II RT Kit. In PCR tubes, a master mix was prepared with the supplied 5× miScript Hispec buffer reaction buffer, 10× miScript Nuleics Mix, miScript reverse transcriptase mix and RNase-free water. Total RNA was added last, in order to achieve a working amount of 1500 ng in a 20 μL volume. The cDNA synthesis was performed according to the following steps: initial step at 37 °C for 60 min and second step, enzyme inactivation, at 95 °C for 5 min. At the end, 310 μL of RNase-free water was added to the 20 μL of cDNA, divided in 3 aliquots of 110 each and stored at −20 °C until further use. Then, the real-time qPCR reaction mix was prepared by adding 1375 μL of 2× QuantiTect SYBR^®^ Green PCR Mastermix buffer, 275 μL of 10× miScript Universal Primer, 1075 μL of RNase-free water and 25 μL of cDNA. From this mixture, 25 μL was added to each well of a 96-well plate containing microRNAs primers. A centrifugation was done for 1 min at ≥1000× *g* at room temperature. The thermal cycler conditions were 15 min at 95 °C, followed by 40 cycles for 15 s at 94 °C, 30 s at 55 °C and 30 s at 70 °C. The relative expression of miRNAs was performed by the comparative 2^−(ΔΔ*C*t)^ method.

### 4.9. Pathway Analysis of miRNA

The DIANA-miRPath v3.0 web server [44] was used for the identification of miRNA target genes using the Kyoto Encyclopedia of Genes and Genomes (KEGG) pathways [45] and gene ontology (GO) terms [46,47]. miRNA target genes were identified using experimentally validated interactions contained in Tarbase v7.0 [44]. The “Genes intersection” method was used for KEGG pathway analysis and GO terms enrichment. An intersection set composed of three miRNAs was used, i.e., all genes targeted by at least three miRNAs contained in the selected set were used for the enrichment analysis. The *p*-value threshold was 0.05. The advanced statistic options included “FDR Correction”.

### 4.10. Statistical Analysis

The data were analyzed with GraphPad Prim^®^ 5.01 for Windows (GraphPad Software, La Jolla CA, USA, www.graphpad.com). Values are presented with an approximate mean ± standard error of the mean (SEM) or mean ± standard error (SD). In the MTT assay, the statistical analysis was performed based on 2-way ANOVA with Bonferroni’s post-test. In the quantification of cellular DiOC2 retention (median fluorescence intensity) by flow cytometry, the statistical analysis was performed based on a one-way ANOVA analysis with Bonferroni’s multiple comparison test. In the analysis of ABCB1 gene expression, the statistical analysis was performed based on an unpaired T-test. A *p*-value < 0.05 was considered statistically significant.

## 5. Conclusions

Taken together, the data suggests that ABCB1 plays a major role in the kinetics of CDR, furthermore, its levels of expression are correlated with miRNAs. We identified a signature of microRNAs expression that might be a good tool to assess loss of drug resistance.

## Figures and Tables

**Table 1:** miRNAs differentially expressed in KCR cells after 16 weeks without DOX, compared to parental KCR cells (week 0). microRNAs were selected by fold-change ≥ 2. Thirteen were downregulated, while ten were overexpressed.

		miRNA Name	Accession Number	Fold-Change
KCR week 16 vs. KCR week 0	Underexpressed	hsa-miR-585-3p	MIMAT0003250	−2.8
		hsa-miR-34a-5p	MIMAT0000255	−3.3
		hsa-miR-877-5p	MIMAT0004949	−3.9
		hsa-miR-1287-5p	MIMAT0005878	−7.0
		hsa-miR-1182	MIMAT0005827	−2.3
		hsa-miR-155-3p	MIMAT0004658	Only expressed in KCR week 0
		hsa-miR-656-3p	MIMAT0003332	−2.1
		hsa-miR-323b-5p	MIMAT0001630	−3.0
		hsa-miR-4304	MIMAT0016854	Only expressed in KCR week 0
		hsa-miR-3691-5p	MIMAT0018120	−4.6
		hsa-miR-676-5p	MIMAT0018203	−2.4
		hsa-miR-4258	MIMAT0016879	Only expressed in KCR week 0
		hsa-miR-3177-3p	MIMAT0015054	−5.3
	Overexpressed	hsa-miR-635	MIMAT0003305	Only expressed in KCR week 16
		hsa-miR-502-5p	MIMAT0002873	4.1
		hsa-miR-342-3p	MIMAT0000753	3.1
		hsa-miR-767-5p	MIMAT0003882	2.3
		hsa-miR-1307-3p	MIMAT0005951	2.7
		hsa-miR-1207-5p	MIMAT0005871	Only expressed in KCR week 16
		hsa-miR-548k	MIMAT0005882	Only expressed in KCR week 16
		hsa-miR-183-3p	MIMAT0004560	4.7
		hsa-miR-1193	MIMAT0015049	Only expressed in KCR week 16
		hsa-miR-187-5p	MIMAT0004561	Only expressed in KCR week 16

**Table 2:** Putative genes targeted by the differentially expressed miRNAs in KCR cells 16 weeks without DOX in the KEGG category “Pathways in Cancer”.

Pathways in Cancer (hsa05200)		
	microRNA ID	Putative Targets
Underexpressed	hsa-miR-34a-5p	CRKL | GNAS | ETS1 | BCL2 | CDK6 | CTNNB1 | E2F3 | LAMC1 | EP300| GNAI2 | CDKN1A
	hsa-miR-877-5p	ITGB1 | CRKL | ETS1 | LAMC1
	hsa-miR-3691-5p	ARNT | ETS1 | EP300
	hsa-miR-155-3p	CDKN1A
Overexpressed	hsa-miR-1307-3p	GNAS | BCL2 | CDK6 | CDKN1A
	hsa-miR-1207-5p	BCL2 | IGF1R | CTNNB1 | EP 300 | GNAI2
	hsa-miR-183-3p	NRAS | CDK6 | CTNNB1 | E2F3
	hsa-miR-767-5p	NRAS | EP300
	hsa-miR-187-5p	ETS1 | IGF1R | CTNNB1
	hsa-miR-635	ITGB1
	hsa-miR-342-3p	ITGB1 | CRKL | GNAS | IGF1R | E2F3 | LAMC1 | EP 300
	hsa-miR-548k	ARNT | CDK6 | CTNNB1 | LAMC1
	hsa-miR-502-5p	NRAS | CRKL | ARNT | GNAI2 | CDKN1A

**Table 3:** Putative genes targeted by the differentially expressed miRNAs in KCR cells, 16 weeks without DOX in the KEGG category “Proteoglycans in Cancer”.

Proteoglycans in Cancer (hsa05205)		
	microRNA ID	Putative Targets
Underexpressed	hsa-miR-155-3p	CDKN1A
	hsa-miR-877-5p	ITGB1
	hsa-miR-34a-5p	THBS1 | CTNNB1 | CDKN1A
Overexpressed	hsa-miR-342-3p	ITGB1 | THBS1 | IGF1R
	hsa-miR-1207-5p	IGF1R | CTNNB1
	hsa-miR-187-5p	IGF1R | CTNNB1
	hsa-miR-1307-3p	THBS1 | CDKN1A
	hsa-miR-635	ITGB1
	hsa-miR-502-5p	NRAS | CDKN1A
	hsa-miR-767-5p	NRAS
	hsa-miR-183-3p	NRAS | CTNNB1
	hsa-miR-548k	CTNNB1

**Table 4:** Putative genes targeted by the differentially expressed miRNAs, in KCR cells 16 weeks without DOX, in the KEGG category “ECM-receptor interaction”.

ECM-Receptor Interaction (hsa04512)		
	microRNA ID	Putative Targets
Underexpressed	hsa-miR-34a-5p	THBS1 | LAMC1
	hsa-miR-877-5p	ITGB1 | LAMC1
Overexpressed	hsa-miR-635	ITGB1
	hsa-miR-548k	LAMC1
	hsa-miR-342-3p	ITGB1 | THBS1 | LAMC1
	hsa-miR-1307-3p	THBS1

**Table 5:** Gene enrichment analysis using GO “Biological Process” terms with genes targeted by the differentially expressed miRNAs between KCR week 0 and KCR week 16. Biological process terms are ordered by increasing *p* value. The table lists number of genes and number of miRNAs in each term.

GO Biological Process Terms	*p*-Value	Genes	miRNAs
cellular nitrogen compound metabolic process	0.000	79	13
biosynthetic process	0.000	60	13
response to stress	0.000	46	13
cellular protein modification process	0.000	37	13
gene expression	0.000	24	11
symbiosis, encompassing mutualism through parasitism	0.000	16	12
mitotic cell cycle	0.000	13	13
viral process	0.000	13	12
blood coagulation	0.000	13	13
immune system process	0.001	29	13
regulation of cell cycle	0.001	11	11
cell cycle arrest	0.001	9	11
Fc-epsilon receptor signaling pathway	0.001	7	10
cell cycle	0.002	21	13
platelet degranulation	0.002	5	9
intrinsic apoptotic signaling pathway	0.004	5	7
transcription, DNA-templated	0.008	39	13
Notch signaling pathway	0.010	8	11
platelet activation	0.010	7	9
neurotrophin TRK receptor signaling pathway	0.010	7	10
cellular response to hypoxia	0.010	7	11
fibroblast growth factor receptor signaling pathway	0.010	7	12
regulation of transcription from RNA polymerase II promoter in response to hypoxia	0.010	3	7
cellular protein metabolic process	0.012	10	13
negative regulation of translation involved in gene silencing by miRNA	0.012	3	6
cell death	0.014	17	12
nuclear-transcribed mRNA catabolic process, deadenylation-dependent decay	0.014	4	9
positive regulation of protein insertion into mitochondrial membrane involved in apoptotic signaling pathway	0.014	3	5
3′-UTR-mediated mRNA stabilization	0.014	3	6
mRNA processing	0.015	13	11
membrane organization	0.015	12	11
DNA methylation	0.015	4	7
positive regulation of nuclear-transcribed mRNA catabolic process, deadenylation-dependent decay	0.015	3	7
organ morphogenesis	0.021	7	11
mRNA splicing, via spliceosome	0.022	8	11
innate immune response	0.026	14	12
positive regulation of nuclear-transcribed mRNA poly(A) tail shortening	0.026	3	7
RNA splicing	0.028	9	11
mRNA metabolic process	0.028	6	9
regulation of translation	0.028	6	9
regulation of mRNA stability	0.029	3	6
chromatin organization	0.031	5	10
PML body organization	0.031	2	5
nucleobase-containing compound catabolic process	0.032	15	12
response to endoplasmic reticulum stress	0.032	5	9
nuclear-transcribed mRNA poly(A) tail shortening	0.033	3	8
epithelial cell differentiation involved in prostate gland development	0.033	2	7
catabolic process	0.034	26	13
epidermal growth factor receptor signaling pathway	0.034	6	10
phosphatidylinositol-mediated signaling	0.034	5	9
intrinsic apoptotic signaling pathway in response to DNA damage by p53 class mediator	0.034	4	9
negative regulation of anoikis	0.034	3	8
regulation of viral genome replication	0.034	2	6
establishment or maintenance of microtubule cytoskeleton polarity	0.042	2	4
negative regulation of transcription from RNA polymerase II promoter	0.043	19	13
T cell differentiation in thymus	0.043	4	6

## Abbreviations

CDR	Cancer drug resistance
DOX	Doxorubicin
